# Telomere-to-Telomere Genome Assembly of *Coprinellus disseminatus* and Genomic Insights into Its Symbiotic Germination of *Cremastra appendiculata* Seeds

**DOI:** 10.3390/jof12070460

**Published:** 2026-06-23

**Authors:** Wenyan Huo, Xuelian He, Jing Su, Lu Dai, Peng Qi, Yu Liu, Liguang Zhang, Ting Qiao, Junzhi Li

**Affiliations:** 1 Fungal Research Center, Shaanxi Provincial Institute of Microbiology, Xi’an 710043, China; huowenyanabace@gmail.com (W.H.); xuelhe@163.com (X.H.); dailutk@126.com (L.D.); qipeng_325@163.com (P.Q.); ly137261323@126.com (Y.L.); liguang_zhang@163.com (L.Z.); syjaqt@163.com (T.Q.); 2 Xi’an Supervision & Inspection Institute of Product Quality, Xi’an 710065, China; su_jing2007@126.com

**Keywords:** *Coprinellus disseminatus*, telomere-to-telomere genome assembly, *Cremastra appendiculata*, symbiotic germination, CAZyme, gene family expansion, orchid mycorrhiza

## Abstract

*Cremastra appendiculata* is a medicinally important orchid whose seed germination depends on fungal symbionts. Here, we present the first telomere-to-telomere (T2T) genome assembly of the orchid mycorrhizal fungus *Coprinellus disseminatus*, comprising 15 gapless chromosomes (54.41 Mb) with 98.80% BUSCO completeness. Symbiotic germination assays demonstrated that *C. disseminatus* significantly outperformed its congeners *C. domesticus* and *C. radians* in protocorm biomass. Comparative genomic analyses revealed highly conserved carbohydrate-active enzyme (CAZyme) repertoires among the three species, ruling out CAZyme divergence as the primary driver of differential symbiotic performance. CAFE analysis showed that since its divergence approximately 117.8 million years ago, *C. disseminatus* underwent substantial gene family expansions enriched in proteasome, endocytosis, adherens junction, and tight junction pathways, suggesting that lineage-specific expansion of these functional modules may have contributed to its superior symbiotic capacity for orchid seed germination. These findings require further experimental validation through transcriptomic and functional genomic approaches.

## 1. Introduction

The Orchidaceae are one of the most species-rich families of angiosperms, containing nearly 28,000 recognized species spread across a broad range of ecological niches around the world [1]. One defining trait of the reproductive biology of orchids is their production of dust-like seeds, which are characterized by minimal endosperm reserves and therefore absolutely require a fungal symbiont to supply carbon and nitrogen during germination [2,3]. In the absence of a compatible fungal symbiont, embryogenesis remains arrested within the germination cycle [4], halting at the stage of embryo imbibition and swelling prior to emergence from the testa [5].

*Cremastra appendiculata* (D. Don) Makino occupies a prominent place within Orchidaceae as a medicinally important species [6], with its native habitat located mainly in southern China [7]. *C. appendiculata* has been widely used in traditional medicine for treating various diseases, and modern clinical research has proven its potent antineoplastic properties [8]. Owing to its promising therapeutic profile, characterized by high efficacy and relatively low systemic toxicity, *C. appendiculata* has been adopted clinically for diverse cancers, including gastric, mammary, lung, liver, and thyroid carcinomas [9,10,11,12,13]. Its anticancer activity is mainly attributed to its pseudobulbs, which are rich in bioactive constituents such as colchicine [8]. Beyond its anticancer effects, it is traditionally used for reducing swelling, dispersing stasis nodules, and clearing toxic heat [14]. *C. appendiculata* also exhibits a broad pharmacological spectrum, including lowering blood sugar, enhancing antioxidant activity, reducing blood pressure, and exerting anti-angiogenic and antimicrobial effects [15,16,17].

Diverging from the accepted orchid mycorrhizal paradigm based on peloton-forming genera such as *Tulasnella*, *Ceratobasidium*, and *Serendipita*, *C. appendiculata* recruited wood-decay saprotrophs of the family *Psathyrellaceae* as its main mycorrhizal partners [18]. This pioneering observation by Yagame and colleagues [18] was the first report of a mycorrhizal symbiosis between a photosynthetic orchid and *Psathyrellaceae* fungi, revealing a previously unsuspected evolutionary pathway towards mycoheterotrophy and broadening our understanding of orchid–fungus relationships. Subsequently, successive studies demonstrated that other *Coprinellus* spp. could also sustain *C. appendiculata* seed germination. Gao and colleagues [19] showed that *C. disseminatus* is particularly efficient, compared with asymbiotic germination, in terms of both germination rate and overall efficiency. During this symbiotic process, fungal mycelia penetrate protocorm tissues, selectively entering cells to form nutrient-releasing pelotons. Further biochemical and transcriptomic analyses revealed that *C. disseminatus* was able to overcome seed coat–imposed dormancy efficiently by digesting 67% and 73% of the lignin content of the seed coat at 6 and 12 days after inoculation, respectively [20]. Based on these findings, *C. radians* has more recently achieved germination rates above 75% under favorable co-cultivation conditions [21]. The fungal symbiont coordinates this transition by employing an arsenal of enzymes, including laccases, cellulases, and xylanases, to degrade the lignified testa of the seed. This enzymatic digestion process not only increases moisture uptake and seed permeability but also creates a favorable internal environment within the seed that facilitates balanced nutrient diffusion and hormonal regulation, both of which are essential for successful seedling emergence [22].

The advent of long-read sequencing technologies, including the ONT ultra-long-read and PacBio high-fidelity (HiFi) sequencing technologies, has enhanced our ability to assemble complete telomere-to-telomere (T2T) genome assemblies with entire chromosome sequences and difficult-to-assemble regions such as centromeres, telomeres, and other complex repeats [23]. This capability gained prominence with the completion of the human T2T genome assembly [24] and has more recently been extended to many plant lineages. In the present study, we employed an integrated sequencing strategy combining ONT ultra-long reads, DNBSEQ short reads, Hi-C chromatin proximity ligation, and transcriptomic sequencing to achieve T2T assembly of *C. disseminatus*. For fungi, the number of T2T genome assemblies has been increasing at a rapidly accelerating pace in recent years. Bowyer et al. [25] recently assembled the first T2T genome for a fungus, *Aspergillus fumigatus*, revealing insights into centromeres and chromosomal rearrangements. Furthermore, Sonnenberg et al. [26] reported T2T completeness for two strains of *Agaricus bisporus*, unveiling polymorphic chromosome termini and offering the first centromere annotation in *Basidiomycota*. Other fungal species now represented by T2T assemblies include *Ganoderma leucocontextum* [27], *Trichoderma simmonsii* [28], and *Rhizoctonia cerealis* [29]. Despite this expanding collection of T2T fungal genomes, no *Coprinellus* species has yet been resolved to T2T completeness.

Our study addresses this knowledge gap by exploiting modern sequencing technologies to construct the first T2T-level genome assembly of *C. disseminatus*. Our primary goal is to provide a high-quality chromosome-level reference genome for this orchid-symbiotic fungus and to characterize its repeats and functional genes. *C. domesticus* and *C. radians* were selected as comparative species because both belong to the genus *Coprinellus*, both have been independently reported to promote *C. appendiculata* seed germination [19,21], and high-quality genome assemblies for these two species were generated in parallel using the same pipeline (see Section 2.6), making them ideal congeners for systematic comparison of symbiotic capacity and genomic architecture. Building upon this genomic resource, we compared *C. disseminatus* with phylogenetically related saprotrophic fungi to trace back their evolutionary history, estimate divergence times, and identify orthologous gene relationships within the family *Psathyrellaceae.* We also characterized the carbohydrate-active enzyme (CAZyme) profiles to better understand lignocellulose-degrading capability. Importantly, we evaluated the capacity of different *Coprinellus* species to induce the germination of *C. appendiculata* seeds and support early growth of the resulting seedlings. Furthermore, we employed gene family expansion analyses along with KEGG pathway enrichment and Gene Ontology (GO) enrichment analyses to elucidate the mechanisms by which *Coprinellus* species evolved the capacity to elicit germination of *C. appendiculata* seeds and promote protocorm development.

## 2. Materials and Methods

### 2.1. Fungal Cultivation and Seed Germination Assay

Three *Coprinellus* species were tested for their effects on *C. appendiculata* seed germination: *C. domesticus* (Cdom), *C. radians* (Crad), and *C. disseminatus* (Cdis).

Flasks 15 cm in diameter were filled with mixed sawdust substrate to approximately 1/4 of the flask height (moisture content was 55% to 60%), which was then sterilized by autoclaving at 121 °C for 2 h. After cooling to room temperature, a mycelial block of each *Coprinellus* species was inoculated into the prepared mixed sawdust, using eight flasks per species. All inoculated bottles were placed on glass shelves at 23 °C to grow mycelia for 5 to 7 days. Approximately 200 surface-sterilized *C. appendiculata* seeds (sodium hypochlorite solution treatment, 10 min) were uniformly sown onto the surface of each fungal culture. The co-cultures were maintained under ambient indoor conditions at 20–25 °C for symbiotic germination.

After the incubation period, all protocorms recovered across the eight flasks of each treatment were pooled, counted, and categorized by developmental stage (protocorm formation only or protocorm with leaf or root emergence). The overall germination rate per treatment was calculated as the total number of protocorms formed divided by the total number of seeds sown across all eight flasks (approximately 200 seeds per flask; 1600 seeds per treatment in total). The fresh weight of every recovered protocorm was measured individually using an analytical balance (Shanghai Precision Scientific Instrument Co., Ltd., Shanghai, China). Because protocorm yields per treatment were very low (5–14 protocorms in total across all eight flasks), flask-level records were insufficient to support mixed-effects modeling; individual protocorm fresh weights were therefore used as the unit of observation. Given that Shapiro–Wilk tests indicated significant departures from normality in two of three groups (*C. radians*: W = 0.742, *p* = 0.001; *C. disseminatus:* W = 0.864, *p* = 0.044), differences in protocorm fresh weight among treatments were assessed using the Kruskal–Wallis test, with pairwise post hoc comparisons performed using the Mann–Whitney U test and Bonferroni correction (adjusted α = 0.0167 for three pairwise comparisons). All statistical analyses were performed in Python (SciPy v1.13). A dot plot overlaid with a box plot was generated to display all individual protocorm fresh weights, with sample sizes (*n* = total protocorms recovered per treatment) annotated for each group.

### 2.2. Fungal Strain and Culture Conditions

*C. disseminatus* was collected from fruiting bodies in Shaanxi Province, China. To obtain sufficient biomass for nucleic acid extraction, purified mycelium from fruiting bodies was cultivated in potato dextrose (PD) liquid medium and then grown in a temperature-controlled orbital shaker (Shanghai Zhicheng Analytical Instrument Manufacturing Co., Ltd., Shanghai, China) at 25 °C for 5–7 days. The actively growing mycelium was collected by filtration through several layers of sterilized gauze and then immediately frozen in liquid nitrogen and stored at −80 °C for nucleic acid extraction. Species identification was confirmed by combined morphological and internal transcribed spacer (ITS) sequence analyses.

### 2.3. Nucleic Acid Extraction and Quality Assessment

Genomic DNA and total RNA were extracted from frozen mycelial material using commercially available kits adapted for fungal material. Nucleic acid purity was assessed spectrophotometrically using a NanoDrop™ instrument (Thermo Fisher Scientific, Waltham, MA, USA) and quantified on a Qubit^®^ 3.0 instrument (Thermo Fisher Scientific). DNA and RNA integrity were further assessed by 1% agarose gel electrophoresis (Beijing Liuyi Biotechnology Co., Ltd., Beijing, China) to verify that sample quality met the requirements for T2T genome assembly library construction.

### 2.4. Library Construction and Sequencing Platform Integration

To obtain ultra-long reads needed for spanning repetitive regions, ONT sequencing (Oxford Nanopore Technologies, Oxford, UK) technology was adopted for ultra-long read generation. High molecular weight genomic DNA was end-repaired and enzymatically converted to single-stranded form to attach sequencing adapters. Nanopore libraries were generated and loaded into R10.4.1 flow cells for real-time sequencing using a PromethION instrument (Oxford Nanopore Technologies, Oxford, UK). Raw signal data were base-called using Dorado (v0.9.0, https://github.com/nanoporetech/dorado, accessed on 28 December 2025) to obtain high-quality ultra-long fragments.

To polish the genome and achieve single-nucleotide accuracy, short-read data were acquired using a DNBSEQ high-throughput sequencer (MGI Tech Co., Ltd., Shenzhen, China). Genomic DNA was fragmented physically or enzymatically, followed by end repair and dA-tailing for adapter ligation. Selective adapter ligation was used to attach adapters to the DNA fragments. Target-size fragments were separated using magnetic bead technology and heat denatured into single-stranded circles for rolling circle amplification. The DNA circles were amplified to generate DNA nanoballs (DNB). Libraries were sequenced in paired-end 150 bp (PE150) mode.

Chromosome-scale scaffolding was based on Hi-C technology that captures genome-wide chromatin spatial interactions. Fresh mycelial cells were fixed using formaldehyde to capture the native chromatin structure and then digested with DpnII restriction endonuclease. Biotin-labeled nucleotides were incorporated during proximity ligation at digestion sites. After removing cross-links and purifying DNA, the samples were digested to a size range of 300–700 bp. Biotinylated fragments from ligation junctions were selectively enriched using streptavidin magnetic beads. Finally, the enriched libraries were sequenced in PE150 mode on a DNBSEQ-T7 sequencer (MGI, Shenzhen, China).

To verify the accuracy of gene predictions and facilitate functional annotation, transcriptome sequencing was performed. Poly(A) + mRNA from total fungal RNA was enriched using oligo-dT magnetic beads. The enriched mRNA was chemically denatured and reverse-transcribed into double-stranded cDNA using random hexamers. The cDNA underwent end repair, dA-tailing at 3′ ends, adapter ligation, and PCR enrichment to generate single-stranded circular DNA libraries. Finally, PE150 sequencing was performed on the DNBSEQ platform (MGI Tech Co., Ltd., Shenzhen, China), acquiring transcriptomic reads for subsequent assembly and genome annotation refinement.

### 2.5. Genome Survey and Preliminary Characterization

Before assembly, preliminary genome attributes were discovered through k-mer frequency distribution analysis. For quality-filtered short reads, k-mer frequency spectra were determined by Jellyfish (v2.2.10) [30] with k = 21, and the frequency spectra were fitted with GenomeScope 2.0 [31] to estimate the genome size, the level of heterozygosity, and the repetitive content of the genome. These estimates informed parameter selection for assembly tools.

### 2.6. Data Quality Control and T2T Assembly Pipeline

After raw sequencing data were filtered, the assembly proceeded. DNBSEQ short reads were trimmed and filtered by fastp (v0.23.2) [32]; Hi-C reads were processed by Trim Galore (v0.6.7) [33]; and ultra-long ONT reads were filtered by Filtlong (v0.2.1) [34] to remove low-quality and ultra-short reads. NECAT (v0.0.1) [35] was used to correct raw ONT reads and produce initial contigs for de novo assembly. These contigs were polished iteratively by Racon (v1.4.3) [36], with ONT reads being realigned to the updated references in each round using minimap2 [37]. For the remaining single-locus errors, short reads were mapped to contigs with BWA-MEM (v0.7.19) [38], sorted and indexed by SAMtools (v1.22.1) [39], and then two more rounds of Pilon (v1.24) [40] were applied for base-level correction.

Chromosome-level scaffolding was obtained by performing Hi-C alignments with Juicer (v1.6) [41] along with 3D-DNA (v180419) [42]. Hi-C contact maps were generated at 100 kb resolutions. Assembly accuracy was checked and refined by manual examination of the Hi-C contact heatmaps in Juicebox Assembly Tools (v2.20.00) [43], where misjoins were detected and corrected, and debris contigs were removed. During this manual curation step, a total of 6 misassemblies were identified and corrected, and 13 small debris scaffolds were removed. The curated assembly was converted into chromosome-scale FASTA sequences using the assembly2agp and agp2fasta modules of CPhasing (v0.2.1) (https://github.com/wangyibin/CPhasing, accessed on 30 December 2025). As the final step, FungalTeloExtender (v1.0.3, https://github.com/huowenyanabace/FungalTeloExtender, accessed on 23 March 2026) was used to detect the telomeric repeat motif and extend telomeric sequences at chromosome termini that lacked sufficient telomeric repeats in the primary assembly. The tool was run with default parameters. It first scans each chromosome end for the presence of the canonical telomeric repeat unit; termini that already contain recognizable telomeric repeat arrays in the primary assembly are recorded as intact and are not subject to extension. Only termini where the telomeric repeat signal is absent or truncated are extended. In the primary assembly, the 5′ terminus of Chr09 already contained a complete telomeric repeat array and therefore required no extension; it is accordingly absent from the extension statistics reported in Table S5, which records only termini that underwent active extension. Extensions were completed for all remaining 29 termini that lacked sufficient telomeric repeats, and all 30 telomeric ends of the 15 chromosomes were confirmed to carry the canonical repeat motif, either pre-existing (Chr09 5′) or newly extended.

The same pipeline, using identical software versions and parameter settings, was applied to generate the genome assemblies of *C. domesticus* and *C. radians*; assembly statistics and quality metrics for all three species are summarized in Table 1. The three assemblies were therefore generated under a uniform workflow and were of comparable quality for downstream comparative analyses. Although their consensus QVs differed (32.98–55.25), all three genomes were resolved as gapless T2T assemblies, had complete telomere-capped chromosome ends, and showed similarly high BUSCO completeness (98.80–99.30%), supporting their use as comparable genomic resources in the orthology, CAZyme, synteny, and gene-family evolution analyses.

### 2.7. Assembly Quality Validation

Assembly quality was also assessed in several dimensions to ensure T2T completeness and accuracy. Gene-space completeness was estimated with BUSCO (v5.4.7) [44] against the fungi_odb10 database in both genome and protein modes. Assembly contiguity metrics were obtained using QUAST (v5.3.0) [45], and the consensus quality value (QV) was determined using Merqury (v1.3) [46] as another measure of base-level quality. Structural correctness of the assembly at the chromosome scale was estimated by generating whole-genome Hi-C contact heatmaps with Juicer Tools [41] and HiCExplorer (v3.6) [47]. Telomere integrity was validated by correlating FungalTeloExtender (v1.0.3, https://github.com/huowenyanabace/FungalTeloExtender, accessed on 23 March 2026) predictions with visual inspection to confirm that each chromosome telomere endpoint would contain recognizable telomeric repeats.

### 2.8. Repetitive Element Annotation

Repetitive sequences were annotated via an integrated de novo and homology-based approach. First, an RMBLAST database was built from the T2T assembly, and then RepeatModeler (v2.0.1) [48] was used to generate a repeat library from the given genome that is specific to that species via a process of iterative discovery and refinement. This custom library, combined with curated sequences from Repbase [49], was passed to the RepeatMasker (v4.2.2) [50] program, which quickly identified and soft-masked repetitive regions genome-wide in sensitive mode. The repetitive landscape was classified into major groups, such as retroelements, DNA transposons, rolling circle elements, simple sequence repeats, low complexity repeats, and small RNA-related sequences.

### 2.9. Gene Prediction and Functional Annotation

Structural gene prediction was performed by combining ab initio algorithms with both transcriptome and homology-based evidence. RNA-seq reads were mapped to the genome with HISAT2 [51] and their respective alignments were submitted as empirical input for a first round of predictions with BRAKER (v2.1.6) [52]. Finally, MAKER (v3.1.3) [53] was used for the final gene prediction and integration. MAKER received four components of evidence: (1) de novo assembled transcripts of the target species from rnaSPAdes (v3.26.0) [54]; (2) de novo transcripts of closely related species; (3) homologous proteins obtained from NCBI databases; and (4) Augustus (v3.5.0) [55] training parameters from the BRAKER step. The MAKER pipeline produced a total of 14,756 gene models, of which 11,737 passed filtering criteria (AED score < 0.5 and minimum protein length of 50 amino acids) and were designated as high-confidence protein-coding genes. This high-confidence set of 11,737 proteins was used for functional annotation. For downstream comparative analyses requiring broader coverage (OrthoFinder, CAFE, CAZyme annotation), the complete predicted protein set of 13,658 sequences (including lower-confidence models passing a relaxed AED threshold of < 1.0) was used to maximize ortholog detection sensitivity. For functional annotation, the 11,737 high-confidence proteins were annotated using eggNOG-mapper (v2.1.12) [56] with GO, KEGG, and COG/KOG terms based on homology-based sequence searches in complete reference databases.

CAZymes were annotated using run_dbcan (v2.0.11) in the dbCAN2 suite [57] using three complementary approaches: HMMER (v3.4) [58] searches against dbCAN HMM profiles (e-value < 1 × 10^−5^, coverage > 0.35); DIAMOND [59] searches against CAZyDB (identity > 30%, coverage > 0.35); and Hotpep (v2.0.11) peptide-based searches [60] (frequency ≥ 2.6, hits ≥ 6). To guarantee annotation reliability and avoid false positives, only genes predicted by at least two of the three methods were retained in the final CAZyme catalog. The CAZymes were then allocated into major categories: Glycoside Hydrolases (GHs), Glycosyl Transferases (GTs), Polysaccharide Lyases (PLs), Carbohydrate Esterases (CEs), Auxiliary Activities (AAs), and Carbohydrate-binding Modules (CBMs).

### 2.10. Genome Visualization and Circos Plot Generation

Whole-genome panoramic maps were plotted with Circos (v0.69-8) [61] to represent integrated and comprehensive views of chromosomal structures and genomic features. From outer to inner ring, Circos concentric tracks displayed chromosome ideograms with size, GC content, gene density, repeat density, and intragenomic synteny ribbons between different chromosomes. The window-based metrics for GC content, gene density, and repeat coverage were estimated using Bedtools (v2.30.0) [62] and seqtk (https://github.com/lh3/seqtk, accessed on 10 March 2026) with properly sized sliding windows to balance resolution and visualization clarity.

### 2.11. Comparative Genomics Framework

Eight fungal species for genomic comparisons were chosen: *C. disseminatus*, two other *Coprinellus* species (*C. domesticus* and *C. radians*, both assembled in this study using the pipeline described in Section 2.6), three other *Psathyrellaceae* species, and two outgroup species (Table S4). Ortholog groups were delimited in a panel of eight species using OrthoFinder (v2.5.4) [63] based on DIAMOND (2.1.17) [59] for rapid similarity searches of sequence files and MAFFT (v7.525) [64] for multiple sequence alignment. Species phylogeny was obtained using concatenation under a maximum-likelihood approach using raxmlHPC-PTHREADS (v8.2.13) [65], as well as coalescent analysis using ASTRAL (v5.7.8) [66].

Divergence time estimation was performed using the MCMCTree program in PAML v4.9 [67]. Molecular dating was calibrated using the *S. cerevisiae–Psathyrellaceae* divergence as the primary calibration point, with a soft minimum bound of 583 MYA and a soft maximum bound of 749 MYA, derived from the TimeTree database [68]. The MCMC analysis was run for 2,000,000 generations with a burn-in of 500,000 generations; convergence was confirmed by an effective sample size (ESS) > 200 for all parameters. Posterior median ages and 95% highest posterior density (HPD) intervals for major nodes are reported in Table S7.

### 2.12. Gene Family Evolution and Functional Enrichment

Dynamics of gene family size evolution were analyzed using CAFE (v4.2.1) [69], which employs a stochastic birth-death process to infer expansions and contractions along each branch of the phylogeny. The global birth-death rate parameter λ was estimated by maximum likelihood using the -lambda_groups option with a pre-specified group structure. Gene families showing significantly accelerated expansion or contraction were identified using a likelihood ratio test with a *p*-value threshold of 0.01. KEGG pathway and GO enrichment analyses of significantly expanded genes were performed with the R package clusterProfiler (v4.18) [70].

### 2.13. Synteny Analysis and Whole-Genome Duplication Detection

Inter- and intra-specific syntenic relationships were identified using JCVI (v1.3.6) [71] through pairwise gene sequence alignment combined with dynamic programming to detect collinear gene blocks. Dot plots were used to visualize macrosynteny and identify chromosomal rearrangements, including inversions, translocations, fusions and fissions. To detect WGD events, synonymous substitution (Ks) distributions were calculated for paralogous pairs (within species) and orthologous pairs (between species) using wgd (v1.1.1) [72]. Ks distribution plots were inspected for characteristic peak patterns indicative of polyploidization, with subsequent analysis distinguishing WGD signatures from tandem or segmental duplications.

### 2.14. Data Visualization and Statistical Software

Data visualization was performed using custom Python (v3.11) and R (v4.5) scripts. All code for data processing and figure generation has been deposited in a public repository (https://github.com/huowenyanabace/T2Tfungi_custom_scripts.git, accessed on 8 April 2026).

## 3. Results

### 3.1. Symbiotic Germination Efficiency of C. appendiculata with Different Coprinellus Species

**Figure 1:**
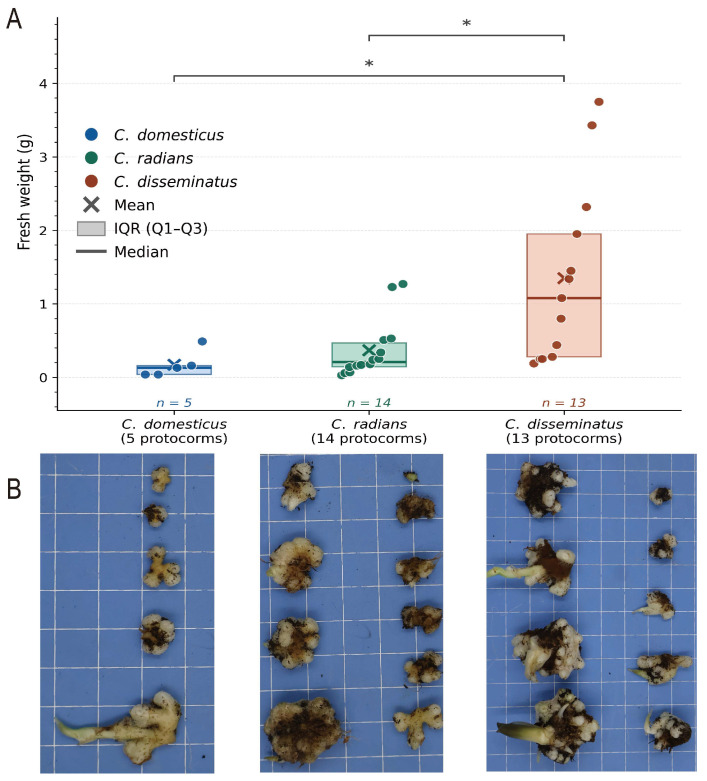
Symbiotic germination efficiency of *Cremastra appendiculata* seeds with three *Coprinellus* species. (**A**) Fresh weight of Cremastra appendiculata protocorms induced by three Coprinellus species. Each point represents one protocorm; boxes indicate the interquartile range (Q1–Q3); horizontal lines indicate medians; × marks indicate means. Statistical comparisons were performed using the Kruskal–Wallis test (H = 12.52, *p* = 0.002), followed by pairwise Mann–Whitney U tests with Bonferroni correction (adjusted α = 0.0167). * *p* < 0.05; ns, not significant. (**B**) Representative photographs of protocorms harvested from each fungal treatment.

Although all three *Coprinellus* species induced protocorm formation in *C. appendiculata*, the efficiency varied considerably among treatments (Figure 1). Kruskal–Wallis analysis confirmed a significant overall difference in protocorm fresh weight among the three treatments (H = 12.52, *p* = 0.002). *C. domesticus* (Cdom) formed 5 protocorms in total across all eight flasks (germination rate: 0.31%), with a mean fresh weight of 0.172 ± 0.186 g (median: 0.130 g; range: 0.04–0.49 g); none of these protocorms developed visible roots or shoots. *C. radians* (Crad) yielded 14 protocorms (germination rate: 0.875%), with a mean fresh weight of 0.370 ± 0.402 g (median: 0.210 g; range: 0.03–1.27 g). *C. disseminatus* (Cdis) also formed 13 protocorms (germination rate: 0.81%) but attained substantially greater biomass, with a mean fresh weight of 1.348 ± 1.208 g (median: 1.080 g; range: 0.19–3.75 g), representing a 7.8-fold and 3.6-fold increase over Cdom and Crad, respectively. Total protocorm biomass across all eight flasks was 0.86 g for Cdom, 5.18 g for Crad, and 17.53 g for Cdis.

Pairwise Mann–Whitney U tests with Bonferroni correction revealed that Cdis protocorms were significantly heavier than those of both Cdom (U = 5.0, *p* = 0.008) and Crad (U = 31.0, *p* = 0.004). No significant difference was detected between Cdom and Crad (U = 19.5, *p* = 0.165). These results demonstrate that *C. disseminatus* produces substantially greater protocorm biomass than its two congeners, establishing it as the most effective *Coprinellus* symbiont for *C. appendiculata* seed germination under the conditions tested.

### 3.2. Sequencing Data Generation and Coverage Statistics

Overall, to build a high-resolution genomic view of *C. disseminatus*, we used a hybrid sequencing strategy combining DNBSEQ, ONT Nanopore and Hi-C technology (Table 2). Our total data yield substantially exceeded the design targets and provided sufficient resolution for obtaining a gapless T2T assembly. Specifically, the DNBSEQ platform enabled us to generate 13.57 Gb of high-quality short-read data, and the ONT Nanopore platform provided 14.68 Gb of effective long-read data (about 258-fold and 284-fold coverage, respectively). We also obtained 19.35 Gb of clean Hi-C data (495× coverage) for scaffolding chromosomes and 34 million clean RNA-seq reads for improving the accuracy of gene structure annotation (Table 2).

### 3.3. T2T Assembly Quality, Structural Validation, and Telomere Integrity

**Figure 2:**
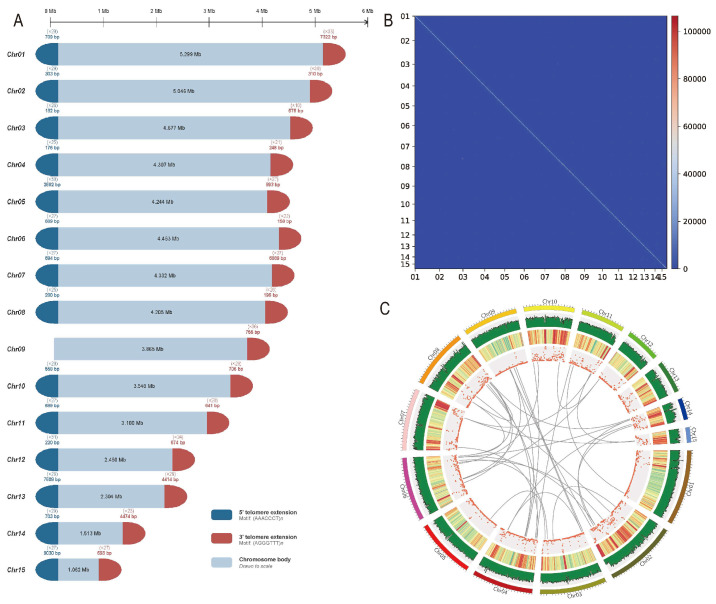
Telomere-to-telomere genome assembly of *C. disseminatus*. (**A**) Chromosome-level ideogram of the 15 gapless chromosomes with 5′ telomere extensions (AAACCCT)n and 3′ telomere extensions (AGGGTTT)n drawn to scale. (**B**) Whole-genome Hi-C contact heatmap at 100 kb resolution confirming chromosome-scale structural integrity. (**C**) Circos plot displaying genomic features from outer to inner rings: chromosome ideograms, GC content, gene density, repeat density, and intragenomic synteny ribbons.

By combining different sequencing datasets, we obtained a continuous, full-length T2T representation of the *C. disseminatus* genome. The final assembly comprised 15 gap-free chromosomes with a total size of 54.41 Mb and a contig N50 of 4.3 Mb (Table 1). The assembly was also evaluated using BUSCO (fungi_odb10) and a rather high completeness score (98.80%) was obtained, validating comprehensive recovery of the gene space (Table 1). High-resolution Hi-C contact maps (100 kb) showed conspicuous diagonal patterns with little off-diagonal noise, effectively eliminating potential large-scale misassemblies such as translocations and inversions (Figure 2B and Figure S1).

All 30 telomeric ends of the 15 chromosomes were confirmed to carry the canonical AAACCCT/AGGGTTT repeat motif, supporting the T2T designation (Table 1). FungalTeloExtender identified this motif and performed targeted extension at 29 chromosome termini where telomeric repeats were absent or truncated in the primary assembly. The 5′ terminus of Chr09 was the sole exception: this end already possessed a complete telomeric repeat array in the primary assembly output and therefore required no extension; it does not appear in Table S5, which records only termini that underwent active extension by FungalTeloExtender. The integrity of all 30 ends was independently verified by visual inspection of terminal sequences in the final assembly. Integration of genome synteny for inter-genome comparisons showed no signature of recent WGD events in *C. disseminatus*, consistent with ploidy stability (Figure 2C).

### 3.4. Repetitive Element Landscapes and Functional Annotation

Analysis of the repetitive sequences using RepeatModeler showed that repeats occupy 14.16% (7.71 Mb) of the genome of *C. disseminatus* (Table 3). Retroelements are the major portion of the repeats, 2966 of which were identified and comprise 3.70 Mb or 6.79% of the total assembly. By contrast, DNA transposons (292 elements, 237 kb, 0.44%) and rolling-circle elements (32 elements, 67.9 kb, 0.12%) are present in the nuclear material but are relatively rare. Meanwhile, a large portion of these integrated repeats is not attributable to any known family (7235 elements, 3.77 Mb, 6.93%). This suggests that lineage-specific repetitive units likely amplified extensively and require further analysis. Additionally, 6538 simple repetitive sequences (304 kb, 0.56%) and 1338 low complexity sequences (76 kb, 0.14%) were identified. Small RNA-containing sequences were also identified at 73 locations covering 206 kb (0.38%).

By integrating transcriptomic data into the annotation pipeline, 11,737 protein-coding loci were annotated across the entire *C. disseminatus* genome (Table 4). Analysis of gene architecture revealed a uniform structure, with an average of five exons per locus, indicating a fairly regular genomic organization throughout the assembly. Orthology analysis assigned 73.56% of the genes to COG functional categories, while pathway analysis mapped 24.92% to KEGG metabolic pathways (Table 4).

### 3.5. Comparative Genomics and Orthologous Group Identification

To understand the evolutionary history and phylogenetic position of *C. disseminatus*, its genome was compared with those of seven other fungal species (Table S4). OrthoFinder identified a total of 9301 orthologous gene families. Among these, approximately 2491 core families were conserved across all eight species, representing the ancestral functional repertoire. At the genus level, 293 orthologous groups were specific to the three *Coprinellus* species, distinguishing this genus from *Coprinopsis* and other *Agaricales*. Given their similar ability to promote orchid seed germination, these gene families likely encode specific functions that evolved or were retained after the divergence of the genus and may be related to orchid symbiosis.

A total of 13,658 proteins were identified in the *C. disseminatus* genome, and 412 corresponding gene families appear to be specific to *C. disseminatus* (Table 5). In comparison, both *C. domesticus* and *C. radians* showed smaller gene sets (11,727 and 12,264 genes, respectively), with fewer unique genes—just 49 families in *C. domesticus* and 90 in *C. radians*. *C. domesticus* and *C. radians* had the most single-copy orthologs among the three species (5767 and 5743), while *C. disseminatus* (5586) also retained a comparable number. The retention of this conserved gene set reflects the close phylogenetic relationship among these species and supports the completeness of our genome assemblies.

### 3.6. Phylogenetic Reconstruction and Divergence Time Estimation

**Figure 3:**
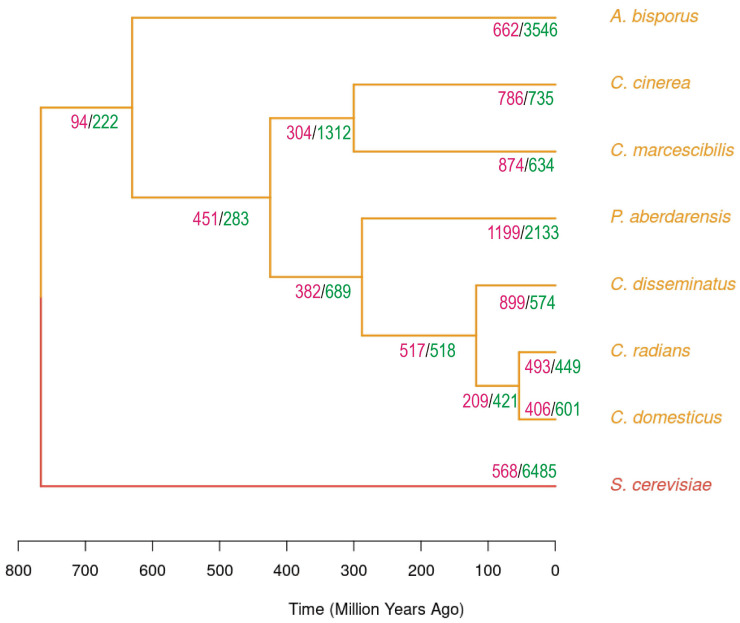
Phylogenetic reconstruction and divergence time estimation of *C. disseminatus* and related fungal species. Time-calibrated phylogeny inferred from single-copy orthologs using maximum likelihood and MCMCTree, with *S. cerevisiae* as outgroup. Numbers at each node indicate gene family expansions (green) and contractions (red) estimated by CAFE analysis. Time scale is in million years ago (MYA).

Using phylogenomic reconstruction based on single-copy orthologs, we obtained a maximum likelihood tree and estimated divergence times using MCMCTree (Figure 3). The topology places *S. cerevisiae* as the root reference and positions *A. bisporus* as an early-branching taxon within *Agaricales*. Among the *Psathyrellaceae*, two lineages are distinguished: one group comprises *C. cinerea* and *C. marcescibilis* in a core *Coprinopsis* group, and the other includes *P. aberdarensis* along with *C. disseminatus*, *C. domesticus*, and *C. radians*.

Molecular dating, anchored by the split between *S. cerevisiae* and *Psathyrellaceae* (~583–749 MYA), recovered the following median divergence times (95% HPD intervals in parentheses; see Table S7 for complete node statistics): the *Agaricales* stem lineage arose around 766.4 MYA; *A. bisporus* diverged from *Psathyrellaceae* around 630.5 MYA; the split between the *Coprinopsis* group and the clade containing *P. aberdarensis* plus three *Coprinellus* species dates to roughly 424.7 MYA; *P. aberdarensis* emerged as a unique lineage near 288 MYA; *C. disseminatus* split from the lineage leading to *C. domesticus* and *C. radians* around 117.8 MYA; and the divergence of *C. domesticus* and *C. radians* occurred around 53.9 MYA. These estimates are broadly consistent with published fungal timescales [68], though direct comparisons are complicated by differences in taxon sampling and calibration strategy.

### 3.7. CAZyme Repertoire and Functional Conservation

Comparative profiling of carbohydrate-active enzymes revealed strong functional coherence among the three *Coprinellus* lineages studied here (Table S1). Total CAZyme numbers were clustered tightly, 493 in *C. disseminatus*, 479 in *C. domesticus,* and 473 in *C. radians*, reflecting a largely conserved enzymatic program with limited divergence since their recent evolutionary split. When this was studied within the six major CAZyme categories, the species showed minimal compositional divergence. GHs dominated the functional program, with almost identical abundances: 168, 169 and 170 genes, respectively. GTs showed only slight differences as well (69, 64 and 63). PLs, CEs and AAs showed highly similar profiles across the three genomes—19/18/18 for PLs, 30/31/30 for CEs and 102/94/93 for AAs. CBMs showed comparable abundances across the three genomes (105/103/99).

Deeper inspection of the family resolution revealed a conserved signature (Table S2). Out of 152 CAZyme families represented in the full comparative dataset, the three focal species presented remarkably conserved distributions. Large cellulolytic families illustrate this conservatism: GH5 ranged narrowly from 21 to 24 copies, GH7 was consistently present at 5 copies per genome, and GH16 ranged from 25 to 27 copies. Oxidative lignin-modifying families matched this too: AA3 varied from 36 to 39 members, and AA9 varied from 13 to 18 across the three genomes.

### 3.8. Gene Family Dynamics and Lineage-Specific Genomic Evolution

**Figure 4:**
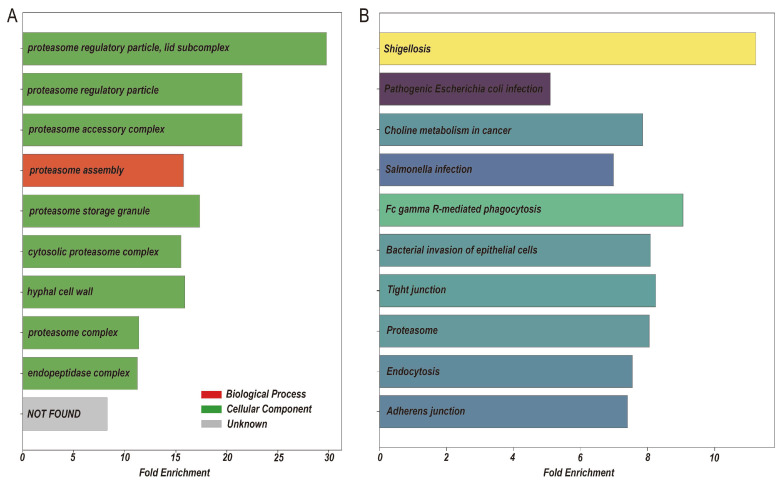
Functional enrichment analysis of expanded gene families in *C. disseminatus*. (**A**) Gene Ontology (GO) enrichment of expanded genes. (**B**) KEGG pathway enrichment of expanded genes.

To reconstruct the evolutionary histories of gene family turnover in the *Coprinellus* clade, we calculated expansion and contraction events along each branch of the phylogenetic tree using CAFE analysis (Figure 3, Table S3). The analysis uncovered major heterogeneity in genome dynamics, with some subclades undergoing extensive repertoire remodeling, whereas others maintained relatively stable repertoires. Among the three focal species, *C. disseminatus* exhibited the most intense evolutionary activity, with 899 gene families undergoing expansion and 574 families undergoing contraction. Of these, 117 families showed significantly accelerated expansion. By contrast, *C. domesticus* and *C. radians* had a more conservative genome architecture: *C. domesticus* showed expansion in 406 families and contraction in 601 families, whereas *C. radians* showed expansion in 493 families and contraction in 449 families. Functional characterization of the 117 significantly expanded *C. disseminatus* families revealed enrichment in cellular organization and protein degradation networks (Figure 4). KEGG pathway analysis identified enrichment in several pathways, including adherens junction, endocytosis, proteasome, and tight junction (Figure 4B). Thus, although *C. domesticus* and *C. radians* also showed branch-specific gene family expansions, the proteasome- and endocytosis-related enrichment discussed here was detected among the significantly expanded families assigned to the *C. disseminatus* branch and is interpreted as a *C. disseminatus*-associated signal rather than a genus-wide *Coprinellus* feature shared equally by all three genomes. The GO profile corroborated these findings: most enriched biological process terms pertained to protein degradation by the proteasome (Figure 4A).

### 3.9. Genomic Synteny and Evolutionary Trajectory Detection

**Figure 5:**
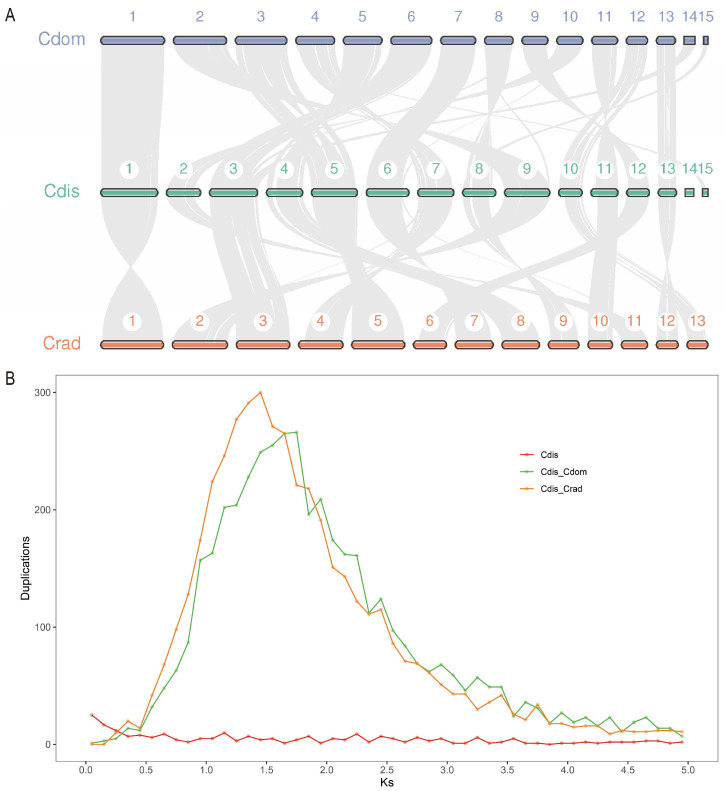
Genomic synteny and whole-genome duplication analysis among three *Coprinellus* species. (**A**) Macrosynteny comparison of *C. domesticus* (Cdom, 15 chromosomes), *C. disseminatus* (Cdis, 15 chromosomes), and *C. radians* (Crad, 13 chromosomes). (**B**) Distribution of synonymous substitution rates (Ks) for paralogous gene pairs within *C. disseminatus* (Cdis) and orthologous pairs between *C. disseminatus* and *C. domesticus* (Cdis_Cdom), as well as *C. disseminatus* and *C. radians* (Cdis_Crad).

To assess the degree of macrosyntenic preservation among the three closely related species, we performed a whole-genome collinearity analysis using JCVI (Figure 5A). Pairwise comparisons revealed extensive syntenic conservation, revealing 8884 orthologous gene pairs between *C. disseminatus* and *C. radians* (Figure S2) and 8862 pairs between *C. domesticus* and *C. disseminatus* (Figure S2). The remarkable density of the collinear gene pairs (65 to 75% of each of these proteomes) again reflected the depth of chromosomal conservation within this clade.

The dot-plot display showed that the chromosomal architectures remained almost unaltered for all three species, and the major diagonal patterns induced by syntenic blocks spanned the length of each chromosome (Figure S2). The 13 chromosomes of *C. radians* mapped precisely onto corresponding regions of the 15 chromosomes in *C. disseminatus*, whereas *C. domesticus* (15 chromosomes) almost exactly aligned with corresponding chromosomes of *C. disseminatus*. A small number of syntenic blocks deviating from the diagonal indicated minor intrachromosomal rearrangements potentially involving inversions or translocations. However, these intrachromosomal rearrangements were rare, involving small chromosomal segments comprising less than 5% of the gene content. The lack of long-range chromosomal reshuffling further supported the evolutionary stability inferred from the phylogenomic and orthology data.

Comparative karyotype mapping among the three species clearly showed one-to-one or one-to-two correspondences between their chromosomes (Figure 5A). This pattern of macrosynteny and minimal gene-order disruption indicated that the chromosomal organization among *C. domesticus*, *C. radians* and *C. disseminatus* remained stable, although they diverged approximately 50 million years ago. To investigate the mechanism of gene family expansion, we examined the distribution of synonymous substitution rates (Ks) to detect potential whole-genome duplication events (Figure 5B). Analysis of paralogous gene pairs in each species did not show a characteristic peak suggestive of ancient polyploidization, implying that tandem duplication or segmental duplication, rather than whole-genome duplication, contributed to the observed repertoire expansions.

## 4. Discussion

In this study, we present the first telomere-to-telomere (T2T) genome assembly of *C. disseminatus* using an integrated multi-platform sequencing approach. Our assembly comprised 15 gapless chromosomes totaling 54.41 Mb, with a contig N50 of 4.3 Mb and a BUSCO completeness score of 98.80%. All 30 telomeric ends were confirmed to carry the canonical AAACCCT/AGGGTTT repeat motif, either pre-existing in the primary assembly or extended by FungalTeloExtender. This complete genome assembly provides the first T2T-level resource for the genus *Coprinellus*, offering a high-quality molecular scaffold for the analysis of repetitive elements, gene content, and regulatory potential.

Symbiotic germination assays confirmed that all three *Coprinellus* species could induce protocorm formation from *C. appendiculata* seeds, but with marked differences in germination efficiency and protocorm biomass. Across 1600 seeds sown per treatment, *C. disseminatus* yielded 13 protocorms (0.81%), *C. radians* yielded 14 (0.88%), and *C. domesticus* yielded only 5 (0.31%). Despite similar total germination counts between *C. disseminatus* and *C. radians*, protocorm biomass differed dramatically: the mean fresh weight per protocorm was 1.348 g for *C. disseminatus*, compared with 0.370 g for *C. radians* and 0.172 g for *C. domesticus*. Kruskal–Wallis analysis confirmed a significant overall difference (H = 12.52, *p* = 0.002), and pairwise Mann–Whitney U tests with Bonferroni correction showed that *C. disseminatus* protocorms were significantly heavier than those of both *C. radians* (*p* = 0.004) and *C. domesticus* (*p* = 0.008). These results reveal *C. disseminatus* as the most effective species in supporting early protocorm development and biomass accumulation in *C. appendiculata* under identical experimental conditions.

Orchid seeds rarely have sufficient endosperm reserves, and seed germination thus largely requires the carbon and nitrogen provided by mycorrhizal symbionts [73]. Previous investigations have established that *C. disseminatus* is capable of dismantling the lignocellulosic barrier within the seed coat of *C. appendiculata* through enzymatic degradation of lignin and cellulose components so as to relieve seed coat dormancy [20]. Furthermore, *C. disseminatus* also elevates endogenous concentrations of plant hormones such as indole-3-acetic acid (IAA), gibberellic acid (GA_3_), salicylic acid (SA), and jasmonic acid (JA), and enhances the activity of antioxidant enzymes in protocorm tissue during symbiosis [74].

To investigate the molecular basis of differential symbiotic capacity, we first compared CAZyme repertoires across the three *Coprinellus* species. The CAZyme catalogs were strikingly similar, suggesting conserved enzymatic profiles since their recent evolutionary divergence. Because *Coprinellus* fungi promote seed germination partly by secreting laccases, cellulases, and xylanases to degrade the lignified seed coat wall [20], CAZyme composition would be expected to correlate with symbiotic performance. However, the homogeneity of the CAZyme arsenals across all three species indicates that differential lignocellulose-degrading capacity is unlikely to be the primary explanation for their contrasting symbiotic efficacies. It is important to note, however, that CAZyme gene content does not capture potential differences in transcript levels, enzyme secretion efficiency, post-translational regulation, or temporal expression patterns, all of which may also contribute to variation in symbiotic effectiveness and warrant future investigation.

CAFE analysis revealed that *C. disseminatus* exhibited greater evolutionary dynamism than its congeners, with the largest number of significantly expanded gene families (117 families, *p* < 0.01). KEGG pathway and GO enrichment analyses indicated that these *C. disseminatus*-expanded genes are enriched in proteasome, endocytosis, adherens junction, and tight junction pathways. Because *C. domesticus* and *C. radians* also contain branch-specific expansions but did not form the enriched gene set highlighted here, we interpret the proteasome- and endocytosis-related signal as associated with the *C. disseminatus* lineage rather than as an equally shared expansion across the three *Coprinellus* genomes. We note that several of these KEGG pathway terms, in particular adherens junctions and tight junctions, are originally defined in the context of animal cell biology, and their application to fungal genomes relies on sequence homology to animal pathway components. In fungi, the annotated genes in these pathways are more accurately interpreted as encoding cell adhesion molecules, cytoskeletal regulators, and signaling components that play analogous roles in hyphal growth and host interaction, rather than the canonical animal junction complexes. The following discussion, therefore, frames these enrichments as biologically plausible hypotheses that are consistent with existing knowledge of orchid mycorrhizal biology but which require experimental validation, particularly through transcriptomic analysis during symbiosis, gene knockout studies, and expression profiling during orchid colonization.

The proteasome constitutes the central executory machinery of the ubiquitin-proteasome pathway, mediating targeted protein turnover and amino acid recycling in mycorrhizal symbiosis [75]. Proteasome-mediated proteolysis may facilitate fungal-to-host nitrogen transfer, where nitrogenous compounds are shuttled as arginine and catabolized to ammonium ions for host translocation [76], providing critical metabolites for embryo development in orchid seeds devoid of endosperm reserves [77]. Transcriptomic evidence from Eucalyptus–Pisolithus ectomycorrhizal systems has confirmed ubiquitin-proteasome pathway upregulation during symbiosis [78], but whether the same holds true in *C. disseminatus–C. appendiculata* interactions remain to be demonstrated.

Endocytosis governs transmembrane nutrient trafficking in mycorrhizal associations [79]. During orchid colonization, extensive plasma membrane invagination envelops developing pelotons, generating amplified symbiotic interfaces [80]. Ultrastructural studies reveal endocytosis-mediated nutrient translocation in Gastrodia–Mycena interactions [81], while arbuscular mycorrhizae require sustained membrane invagination for periarbuscular membrane formation [80,82]. Expansion of endocytosis-related gene families in *C. disseminatus* may therefore reflect an enhanced capacity for nutrient exchange at the symbiotic interface, though direct evidence from this system is lacking.

Although adherens and tight junction pathway terms originate from animal cell definitions, the fungal gene families annotated to these pathways in *C. disseminatus* encompass putative cell adhesion molecules and cytoskeletal regulators that may be instrumental in plant–fungal interactions [83,84]. Adhesion likely constitutes a prerequisite for successful colonization, requiring firm hyphal attachment before enzymatic testa penetration [20]. Tight junction-associated signaling components may modulate the transduction of mycorrhizal factors and effector proteins [85]. These remain speculative assignments, and their functional relevance in the *C. disseminatus–C. appendiculata* system requires direct experimental confirmation.

The molecular clock estimation placed the divergence of *C. disseminatus* from the common ancestor of *C. domesticus* and *C. radians* at approximately 117.8 MYA (95% HPD: see Table S7). Over this period of independent evolution, *C. disseminatus* underwent substantial gene family expansion in the pathways described above. These expansions may have contributed to the development of a distinctive genetic background that facilitated co-evolution with *C. appendiculata*, but causality cannot be inferred from comparative genomic data alone. Future studies integrating transcriptomics during active symbiosis, functional gene knockouts, and comparative analyses with non-symbiotic *Coprinellus* strains will be essential to determine which of these expanded gene families are mechanistically important for orchid mycorrhizal competence.

## 5. Conclusions

Our study offers the first telomere-to-telomere (T2T) genome assembly of the orchid mycorrhizal fungus *C. disseminatus*, filling a critical gap in T2T-level genomic resources for the genus *Coprinellus*. The assembled genome comprises 15 gapless chromosomes with a total size of 54.41 Mb and a BUSCO completeness of 98.80%, providing a high-fidelity reference platform for downstream comparative genomic and functional investigations. Through a systematic side-by-side comparative study of the mycorrhizal germination capacity of three *Coprinellus* species with *C. appendiculata* seeds under uniform conditions, this work demonstrates for the first time that *C. disseminatus* significantly outperforms its two congeners in protocorm biomass production, establishing it as the most effective species for mycorrhizal-based artificial propagation and germplasm conservation of this orchid species.

Comparative CAZyme profiling revealed high functional conservation in carbohydrate-degrading capacity among the three *Coprinellus* species, effectively ruling out CAZyme divergence as the primary driver of differential symbiotic germination performance. Gene family evolution analysis showed that since *C. disseminatus* diverged from the ancestor of the *C. domesticus–C. radians* clade approximately 117.8 MYA, it has experienced substantial gene family expansion in functional modules, including the proteasome, endocytosis, adherens junction, and tight junction pathways. These findings suggest that selective gene family expansion in these pathways may have contributed to the superior symbiotic capacity of *C. disseminatus*, though the underlying mechanisms remain to be validated through transcriptomic and functional genomic approaches.

## Figures and Tables

**Table 1:** Genome Assembly Statistics.

Feature	*C. disseminatus*	*C. domesticus*	*C. radians*
Assembly level	T2T	T2T	T2T
Total genome size (Mb)	54.41	38.57	46.01
Chromosome number	15	15	13
Largest chromosome (Mb)	5.30 (Chr01)	4.59 (Chr01)	4.79 (Chr01)
Smallest chromosome (Mb)	1.06 (Chr15)	0.48 (Chr15)	2.33 (Chr12)
Gap number	0	0	0
GC content (%)	54%	53%	53%
N50	4,306,587 bp	2,975,136 bp	4,185,136 bp
N90	2,450,142 bp	1,879,067 bp	2,438,444 bp
L50	6	6	6
L90	12	12	12
QV	32.98	55.25	40.81
Telomere motif	AAACCCT	TTAGGG	TTAGGG
Telomere-capped ends	30/30 (100%)	30/30 (100%)	26/26 (100%)
BUSCO (fungi_odb10)	98.80%	99.20%	99.30%
NCBI/GSA Accession	PRJNA1450334/PRJCA038423	PRJNA1450334/PRJCA038423	PRJNA1450334/PRJCA038423

**Table 2:** Sequencing Data Statistics.

Species	Data Type	Total Base of Raw Data (Gb)	Total Base of Clean Data (Gb)	Total Reads of Raw Data	Total Reads of Clean Data	Coverage (X)
*Coprinellus disseminatus*	Short-read WGS	~14.02	~13.57	93.45 M	92.65 M	258 X
*Coprinellus disseminatus*	Long-read	~15.48	~14.68	742,469	697,877	284 X
*Coprinellus disseminatus*	Hi-C	~19.38	~19.35	64,585,848	64,585,848	495 X
*Coprinellus disseminatus*	RNA-seq	~10.17	/	33,895,056	/	/

**Table 3:** Repeat Element Statistics.

Element Type	Number of Elements	Length (bp)	Percentage of Sequence (%)
Retroelements	2966	3,696,090	6.79%
DNA transposons	292	237,335	0.44%
Rolling-circles	32	67,931	0.12%
Unclassified	7235	3,772,279	6.93%
Total interspersed repeats	/	7,705,704	14.16%
Small RNA	73	206,431	0.38%
Simple repeats	6538	304,399	0.56%
Low complexity	1338	76,104	0.14%

**Table 4:** Gene Annotation Statistics.

Feature	*Coprinellus disseminatus*
Total gene models (pre-filtering)	14,756
High-confidence protein-coding genes (AED < 0.5)	11,737
Proteins used for comparative analyses (AED < 1.0)	13,658
Average gene length (bp)	2110.68
Average exons per gene	5.6
Average intron length (bp)	71.42
Genes with GO annotation (%)	31.83%
Genes with COG annotation (%)	73.56%
Genes with KEGG KO annotation (%)	40.09%
Genes with KEGG pathway annotation (%)	24.92%

**Table 5:** Ortholog Statistics.

Species	Genes	Species-Specific	Single-Copy
*C. domesticus*	11,727	49	5767
*C. radians*	12,264	90	5743
*C. disseminatus*	13,658	412	5586
*C. cinerea*	11,849	655	5457
*C. marcescibilis*	13,274	697	5458
*P. aberdarensis*	13,898	938	4620
*A. bisporus*	10,095	1732	4754
*S. cerevisiae*	4367	660	2620

## Data Availability

The assembled genome sequences are publicly accessible through the National Center for Biotechnology Information (NCBI) under the BioProject accession PRJNA1450334. Raw sequencing reads have been archived in the Genome Sequence Archive (GSA), hosted by the China National Center for Bioinformation (CNCB), with the corresponding BioProject identifier PRJCA038423. All custom scripts developed for data analysis and figure generation are freely available via GitHub at https://github.com/huowenyanabace/T2Tfungi_custom_scripts.git (v1.0.0).

## Supplementary Materials

The following supporting information can be downloaded at: https://www.mdpi.com/article/10.3390/jof12070460/s1. Figure S1: Per-chromosome Hi-C contact heatmaps for all 15 chromosomes of the *C. disseminatus* T2T genome assembly; Figure S2: Dot-plot analysis of pairwise whole-genome synteny between *Coprinellus* species; Table S1: Statistics of CAZyme gene numbers; Table S2: Detailed comparison of major CAZyme families; Table S3: Gene Family Evolution Summary; Table S4: Species used for comparative genomics analysis; Table S5: Detailed Extension Statistics of *C. disseminatus*; Table S6: Raw Germination Data; Table S7: Divergence Time Estimates with 95% HPD Intervals; Table S8: Detailed repeat element family composition of the *C. disseminatus* T2T genome.
