# Supplementary material for: Telomere-to-Telomere Genome Assembly of Coprinellus disseminatus and Genomic Insights into Its Symbiotic Germination of Cremastra appendiculata Seeds
Source: J Fungi (Basel). 2026 Jun 23;12(7):460. doi: 10.3390/jof12070460 (PMC13412209; doi:10.3390/jof12070460)
Supplement: Supplementary file 1 [file jof-12-00460-s001.zip › jof-4292447-supplementary.pdf]

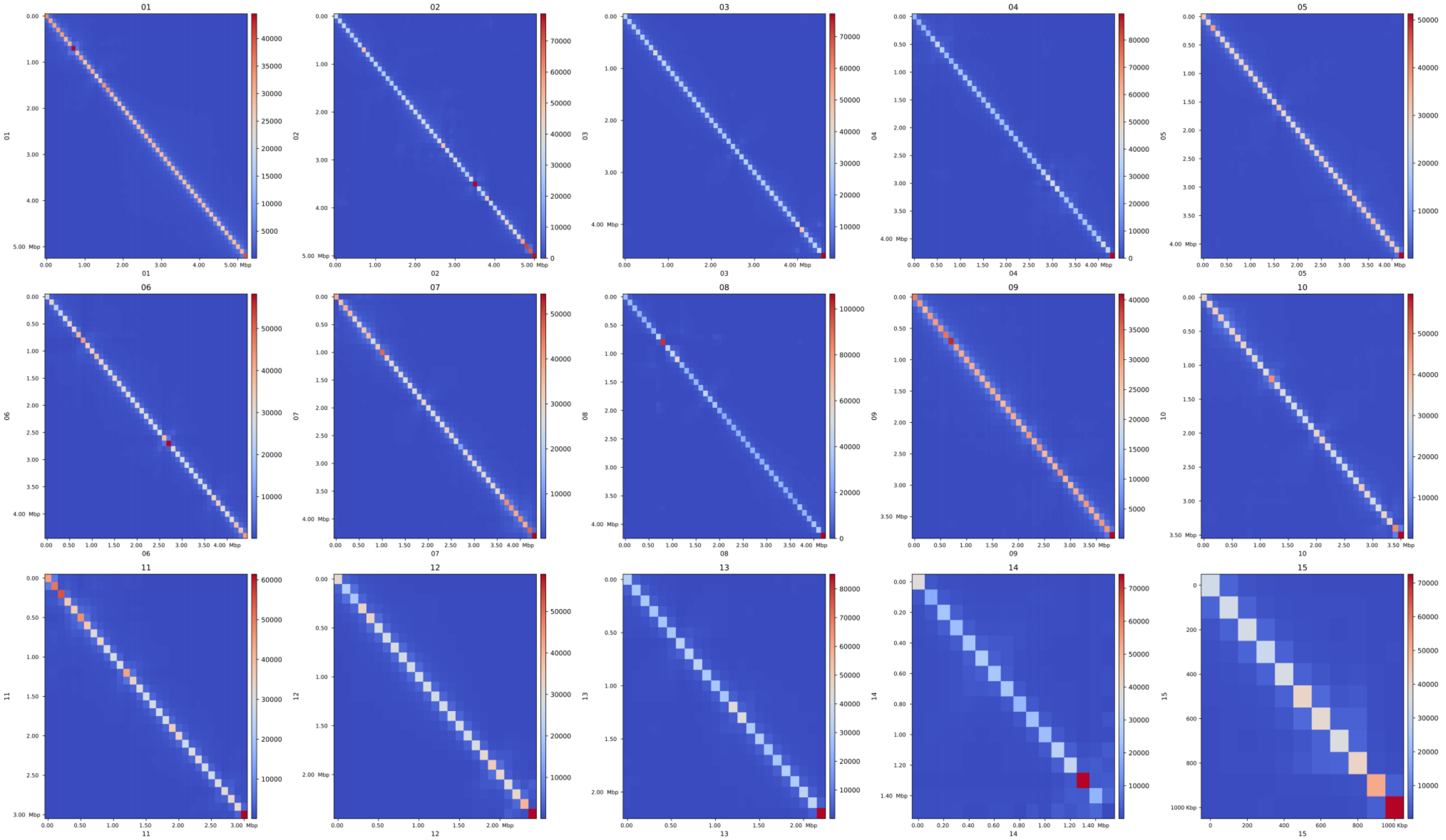

Figure S1. Per-chromosome Hi-C contact heatmaps for all 15 chromosomes of the *C. disseminatus* T2T genome assembly.

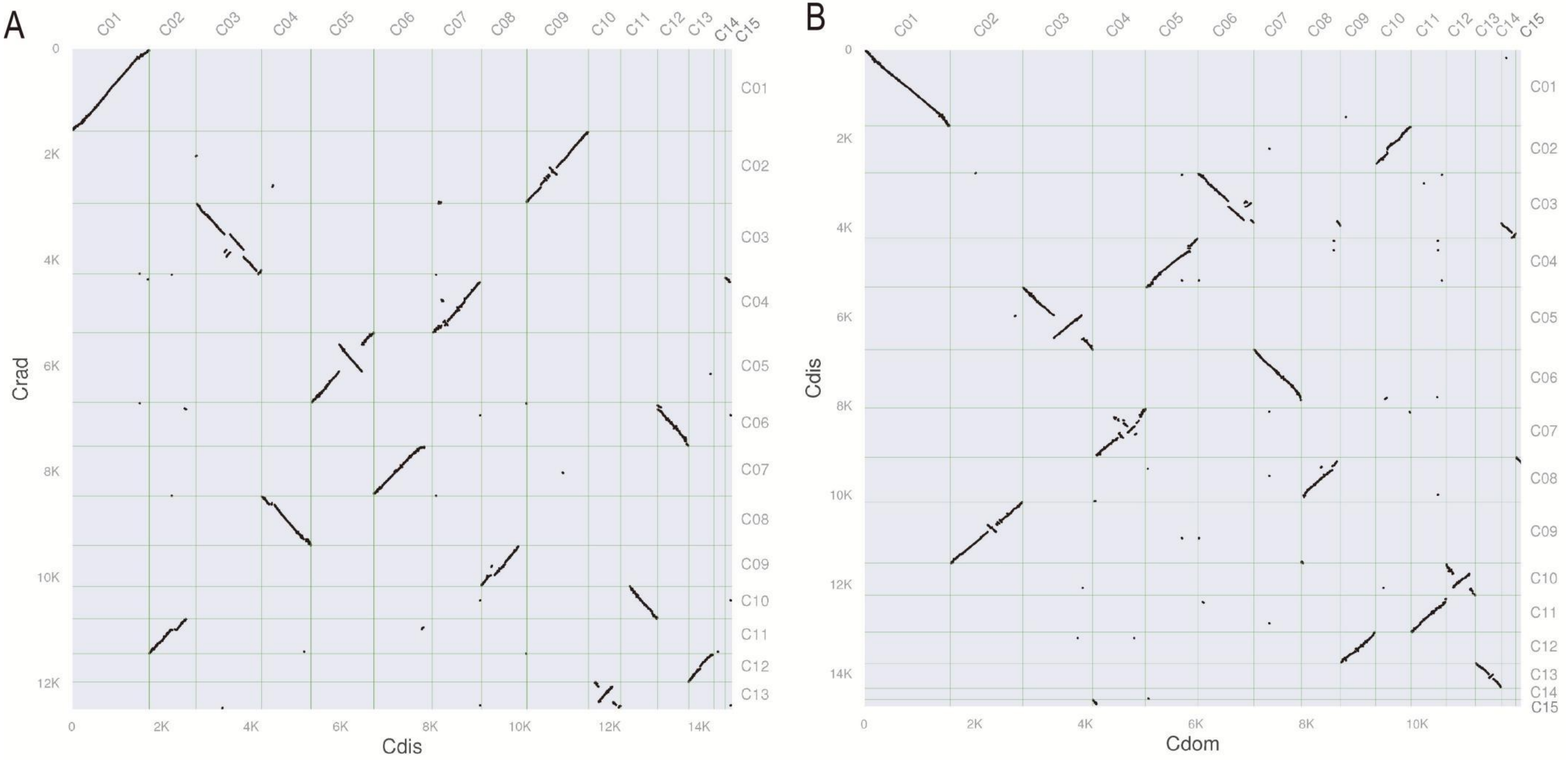

Figure S2. Dot-plot analysis of pairwise whole-genome synteny between *Coprinellus* species. (A) Dot plot of *C. disseminatus* (Cdis) versus *C. radians* (Crad). (B) Dot plot of *C. domesticus* (Cdom) versus *C. disseminatus* (Cdis)

Table S1. Statistics of CAZyme gene numbers

**CAZyme Class**	Abis	Ccin	Cdis	Cdom	Crad	Cmar	Paber	Scere
Glycoside Hydrolases (GH)	165	172	168	169	170	157	174	58
Glycosyl Transferases (GT)	58	69	69	64	63	69	66	68
Polysaccharide Lyases (PL)	12	19	19	18	18	15	17	0
Carbohydrate Esterases (CE)	19	39	30	31	30	37	38	2
Auxiliary Activities (AA)	74	98	102	94	93	102	127	7
Carbohydrate-Binding Modules (CBM)	66	114	105	103	99	95	113	20
Total CAZymes	394	511	493	479	473	475	535	155

Table S2. Detailed comparison of major CAZyme families

CAZyme Family	Abis	Ccin	Cdis	Cdom	Crad	Cmar	Paber	Scere
AA0	0	1	1	1	1	0	1	0
AA1	13	17	19	9	11	20	18	3
AA2	3	3	3	4	4	8	3	1
AA3	34	33	36	39	39	28	56	0
AA5	9	6	5	6	5	7	7	0
AA6	4	3	5	3	3	3	2	3
AA7	0	0	1	3	3	0	1	0
AA8	1	8	7	7	7	9	8	0
AA9	8	19	18	15	13	17	23	0
AA12	1	7	6	6	6	8	7	0
AA14	1	1	1	1	1	2	1	0
CBM0	0	0	2	1	0	0	0	0
CBM1	27	58	58	52	52	50	65	1
CBM2	5	14	17	17	14	10	17	0
CBM3	0	1	0	1	0	0	1	0
CBM4	0	2	2	0	0	1	0	0
CBM5	5	2	2	2	3	2	3	0
CBM6	4	1	2	3	2	0	2	0
CBM10	0	1	0	0	0	0	1	0
CBM13	4	6	4	5	6	3	3	0
CBM16	0	1	1	1	1	1	1	0
CBM18	3	8	7	8	9	8	8	2
CBM19	3	1	1	0	0	0	0	1
CBM20	4	6	3	3	3	3	3	2
CBM21	0	2	0	0	1	2	0	4
CBM22	0	2	0	3	1	1	1	0
CBM24	2	1	1	0	0	3	2	0
CBM25	1	0	1	1	1	0	1	0
CBM27	0	1	0	0	0	2	0	0
CBM32	0	1	1	1	1	1	1	0
CBM35	2	1	1	1	1	1	1	0
CBM38	0	0	0	0	0	0	0	1
CBM39	0	1	0	0	0	1	0	0
CBM43	1	2	1	1	1	5	1	3
CBM48	1	1	1	1	1	1	1	6
CBM50	2	0	0	0	0	0	0	0
CBM51	2	0	0	0	0	0	0	0
CBM60	0	1	0	2	1	0	1	0
CBM61	0	0	0	0	1	0	0	0
CE0	0	2	0	0	0	2	3	0
CE1	1	4	3	3	3	4	7	0
CE2	0	1	0	0	0	1	0	0

CE3	0	1	3	2	1	3	2	0
CE4	6	13	11	11	12	13	10	2
CE5	5	6	1	0	0	0	1	0
CE8	1	0	0	0	0	0	0	0
CE9	1	1	1	1	1	1	1	0
CE12	2	1	1	1	1	1	1	0
CE15	1	7	7	8	8	9	10	0
CE16	1	2	2	4	3	2	2	0
CE17	1	1	1	1	1	1	1	0
GH0	3	3	1	6	4	2	5	5
GH1	2	2	3	2	3	2	2	0
GH2	2	2	2	2	2	2	2	0
GH3	8	7	7	6	6	5	6	0
GH5	18	24	21	22	24	27	32	5
GH6	1	5	2	3	3	3	5	0
GH7	1	6	5	5	5	4	5	0
GH9	1	1	1	1	1	1	1	0
GH10	2	5	7	7	8	4	6	0
GH11	2	4	3	4	2	2	5	0
GH12	2	0	0	0	0	0	0	0
GH13	7	9	7	7	7	8	6	9
GH15	2	4	2	2	2	2	2	1
GH16	20	26	26	25	27	23	21	5
GH17	2	2	3	3	3	2	2	4
GH18	12	9	8	9	8	9	10	2
GH20	4	2	2	2	2	2	3	0
GH25	4	0	1	0	0	0	0	0
GH27	4	0	1	1	0	0	1	0
GH28	3	2	4	2	3	2	2	1
GH29	1	0	0	0	0	0	0	0
GH30	3	3	3	3	4	3	2	0
GH31	6	3	2	2	2	2	2	1
GH32	0	0	0	0	0	0	0	1
GH35	1	0	2	1	2	1	2	0
GH37	2	2	2	2	2	2	3	2
GH38	1	1	1	1	1	1	1	1
GH43	4	4	5	7	6	3	2	0
GH44	1	1	1	1	1	1	0	0
GH47	6	8	7	7	7	6	7	3
GH51	1	1	1	1	1	1	1	0
GH53	1	1	1	1	1	1	1	0
GH55	1	1	3	3	3	2	3	0
GH62	0	3	2	2	1	2	3	0
GH63	2	2	2	1	1	2	1	2

GH65	0	0	0	0	0	0	0	1
GH71	2	4	3	3	3	4	4	0
GH72	1	2	1	1	1	5	1	5
GH74	1	1	1	1	1	1	1	0
GH76	1	0	0	1	1	0	0	2
GH78	4	0	0	0	0	0	0	0
GH79	4	3	7	5	5	2	6	0
GH81	0	0	0	0	0	0	0	2
GH85	1	2	1	1	1	2	1	0
GH88	1	1	1	1	1	1	0	0
GH92	5	3	3	3	2	2	3	0
GH95	1	0	0	0	0	0	0	0
GH105	3	2	0	1	1	1	1	0
GH115	2	1	1	1	1	1	3	0
GH125	1	1	1	1	1	1	1	0
GH128	0	2	2	1	2	2	1	0
GH131	1	1	1	1	1	1	1	0
GH132	0	0	0	0	0	0	0	5
GH133	1	1	1	1	1	1	1	1
GH145	1	1	1	1	1	1	1	0
GH152	3	3	5	4	4	4	4	0
GH154	1	1	1	1	1	1	1	0
GH162	1	0	0	0	0	0	0	0
GT0	2	3	3	3	3	3	3	0
GT1	2	3	1	1	1	2	1	3
GT2	13	15	16	16	16	16	19	5
GT3	1	1	1	1	1	1	1	2
GT4	4	4	4	4	4	4	2	3
GT5	1	1	1	1	1	1	1	0
GT8	1	2	2	2	1	1	2	3
GT15	3	5	4	2	3	8	3	9
GT17	1	0	1	1	1	0	2	0
GT20	3	3	3	3	3	3	3	4
GT21	1	1	1	1	1	1	1	0
GT22	3	4	3	3	3	4	3	4
GT24	1	1	1	1	1	1	1	1
GT31	1	1	1	1	1	1	1	0
GT32	1	1	1	1	1	1	1	4
GT33	1	1	1	1	1	1	2	1
GT34	0	0	0	0	0	0	0	2
GT35	1	1	1	2	1	1	1	1
GT39	3	3	3	3	3	3	3	7
GT48	2	2	6	2	2	2	2	3
GT49	1	1	1	1	1	0	1	0

GT50	1	1	1	1	1	1	1	1
GT57	2	2	2	2	2	2	2	2
GT58	1	1	1	1	1	1	1	1
GT59	1	1	1	1	1	1	0	1
GT62	0	0	0	0	0	0	0	3
GT66	1	1	1	1	1	1	1	1
GT69	2	3	2	2	2	2	3	0
GT71	0	0	0	0	0	0	0	6
GT76	0	0	0	0	0	0	0	1
GT90	4	7	6	6	6	7	5	0
PL1	2	1	2	1	1	0	1	0
PL3	1	2	1	1	1	2	2	0
PL4	1	2	2	2	2	1	2	0
PL8	1	1	2	2	2	1	1	0
PL14	3	7	7	7	7	6	5	0
PL26	1	0	0	0	0	0	0	0
PL35	1	3	2	2	2	2	4	0
PL38	1	2	2	2	2	2	1	0
PL42	1	1	1	1	1	1	1	0

Table S3. Gene Family Evolution Summary

Species/Node	Expansions	Genes Gained	Equal	Contractions	Genes Lost	Families Lost	Avg Expansion	Rapid Expansions	Rapid Contractions	Total Rapid Changes
Cdis<6>	899	2197	7828	574	657	382	0.165574	117	20	137
<11>	304	534	7685	1312	1341	1247	-0.0867649	26	7	33
Paber<8>	1199	4317	5969	2133	2255	1744	0.221697	125	3	128
Crad<4>	493	977	8359	449	634	222	0.0368778	62	51	113
Cdom<2>	406	670	8294	601	864	280	-0.020858	46	87	133
Abis<14>	662	2742	5093	3546	3898	3111	-0.124288	30	5	35
Scere<0>	568	867	2248	6485	7852	5989	-0.750995	2	27	29
<5>	517	981	8266	518	699	359	0.0303193	63	17	80
<3>	209	328	8671	421	525	243	-0.0211805	21	19	40
<7>	382	819	8230	689	719	627	0.0107515	67	0	67
<13>	94	129	8985	222	230	215	-0.010859	0	2	2
Ccin<12>	786	2025	7780	735	1105	472	0.0989141	45	21	66
<9>	451	849	8567	283	364	250	0.0521449	44	9	53
Cmar<10>	874	3164	7793	634	819	438	0.252123	97	14	111

Note: Nodes with only numbers (e.g., <11>, <13>) represent ancestral internal nodes in the phylogenetic tree. CAFE labeled tree: (Scere<0>,(((((Cdom<2>,Crad<4>)<3>,Cdis<6>)<5>,Paber<8>)<7>,(Cmar<10>,Ccin<12>)<11>)<9>,Abis<14>)<13>)<1>

Table S4. Species used for comparative genomics analysis

Species	Abbreviation	Lifestyle	Genome Source
Coprinellus domesticus	Cdom	Saprotrophic	This study
Coprinellus radians	Crad	Saprotrophic	This study
C.disseminatus	Cdis	Saprotrophic	This study
Coprinopsis cinerea	Ccin	Saprotrophic	Ensemblgenomes
Coprinopsis marcescibilis	Cmar	Saprotrophic	Ensemblgenomes
Psathyrella aberdarensis	Paber	Saprotrophic	Ensemblgenomes
Agaricus bisporus	Abis	Saprotrophic	Ensemblgenomes
Saccharomyces cerevisiae	Scere	Outgroup	Ensemblgenomes

Table S5. Detailed Extension Statistics of *C.disseminatus*

Species	Chromosome	Terminus	Extension Length (bp)	Motif	Repeat Count
C.disseminatus	Chr01	3prime	7,322	AGGGTTT	33
C.disseminatus	Chr01	5prime	709	AAACCCT	29
C.disseminatus	Chr02	3prime	310	AGGGTTT	30
C.disseminatus	Chr02	5prime	303	AAACCCT	29
C.disseminatus	Chr03	3prime	676	AGGGTTT	16
C.disseminatus	Chr03	5prime	182	AAACCCT	26
C.disseminatus	Chr04	3prime	248	AGGGTTT	21
C.disseminatus	Chr04	5prime	176	AAACCCT	25
C.disseminatus	Chr05	3prime	693	AGGGTTT	27
C.disseminatus	Chr05	5prime	2,682	AAACCCT	30
C.disseminatus	Chr06	3prime	158	AGGGTTT	22
C.disseminatus	Chr06	5prime	689	AAACCCT	27
C.disseminatus	Chr07	3prime	6,889	AGGGTTT	27
C.disseminatus	Chr07	5prime	694	AAACCCT	27
C.disseminatus	Chr08	3prime	196	AGGGTTT	26
C.disseminatus	Chr08	5prime	280	AAACCCT	25
C.disseminatus	Chr09	3prime	755	AGGGTTT	36
C.disseminatus	Chr10	3prime	706	AGGGTTT	29
C.disseminatus	Chr10	5prime	558	AAACCCT	29
C.disseminatus	Chr11	3prime	641	AGGGTTT	20
C.disseminatus	Chr11	5prime	689	AAACCCT	27
C.disseminatus	Chr12	3prime	674	AGGGTTT	24
C.disseminatus	Chr12	5prime	220	AAACCCT	31
C.disseminatus	Chr13	3prime	4,414	AGGGTTT	29
C.disseminatus	Chr13	5prime	7,609	AAACCCT	28
C.disseminatus	Chr14	3prime	4,474	AGGGTTT	23
C.disseminatus	Chr14	5prime	703	AAACCCT	29
C.disseminatus	Chr15	3prime	693	AGGGTTT	27
C.disseminatus	Chr15	5prime	9,030	AAACCCT	27

Table S6. Raw Germination Data

Protocorm ID	Treatment	Seeds Sown (total)	Individual Fresh Weight (g)
Cdom-01	*C. domesticus*	1,600	0.49
Cdom-02	*C. domesticus*	1,600	0.16
Cdom-03	*C. domesticus*	1,600	0.13
Cdom-04	*C. domesticus*	1,600	0.04
Cdom-05	*C. domesticus*	1,600	0.04
Crad-01	*C. radians*	1,600	1.27
Crad-02	*C. radians*	1,600	0.34
Crad-03	*C. radians*	1,600	0.07
Crad-04	*C. radians*	1,600	0.06
Crad-05	*C. radians*	1,600	0.03
Crad-06	*C. radians*	1,600	1.23
Crad-07	*C. radians*	1,600	0.51
Crad-08	*C. radians*	1,600	0.53
Crad-09	*C. radians*	1,600	0.24
Crad-10	*C. radians*	1,600	0.25
Crad-11	*C. radians*	1,600	0.16
Crad-12	*C. radians*	1,600	0.14
Crad-13	*C. radians*	1,600	0.17
Crad-14	*C. radians*	1,600	0.18
Cdis-01	*C. disseminatus*	1,600	0.19
Cdis-02	*C. disseminatus*	1,600	0.8
Cdis-03	*C. disseminatus*	1,600	1.34
Cdis-04	*C. disseminatus*	1,600	1.08
Cdis-05	*C. disseminatus*	1,600	0.28
Cdis-06	*C. disseminatus*	1,600	3.75
Cdis-07	*C. disseminatus*	1,600	0.25
Cdis-08	*C. disseminatus*	1,600	1.45
Cdis-09	*C. disseminatus*	1,600	3.43
Cdis-10	*C. disseminatus*	1,600	0.44
Cdis-11	*C. disseminatus*	1,600	1.95
Cdis-12	*C. disseminatus*	1,600	0.25
Cdis-13	*C. disseminatus*	1,600	2.32

Note: Flask-level records were consolidated prior to data collection; individual flask identities are unavailable.

Table S7. Divergence Time Estimates with 95% HPD Intervals

Node	Comparison	Median Age (MYA)	95% HPD Lower (MYA)	95% HPD Upper (MYA)
Root	*S. cerevisiae vs Psathyrellaceae*	766.4	641.1	855.8
Node 9	*A. bisporus vs Psathyrellaceae*	630.5	573.5	724.6
Node 7	*Coprinopsis group vs (P. aberdarensis + Coprinellus)*	424.7	329.8	555.6
Node 6	*C. marcescibilis + C. cinerea split*	300.2	206.8	405.1
Node 5	*P. aberdarensis divergence*	288	175.2	401.6
Node 3	*C. disseminatus vs (C. domesticus + C. radians)*	117.8	49.8	173.1
Node 1	*C. domesticus vs C. radians*	53.9	19.3	88.8

Table S8. Detailed repeat element family composition of the C. disseminatus T2T genome

Category	Subcategory	Family	Number of Elements	Length (bp)	Percentage of Sequence (%)
Retroelements	—	Total Retroelements	2966	3696090	6.79
Retroelements	SINEs	Total SINEs	19	5818	0.01
Retroelements	LINEs	Total LINEs	325	579054	1.06
Retroelements	LINEs	CRE/SLACS	0	0	0
Retroelements	LINEs	L2/CR1/Rex	0	0	0
Retroelements	LINEs	R1/LOA/Jockey	0	0	0
Retroelements	LINEs	R2/R4/NeSL	0	0	0
Retroelements	LINEs	RTE/Bov-B	0	0	0
Retroelements	LINEs	L1/CIN4	0	0	0
Retroelements	LTR elements	Total LTR elements	2622	3111218	5.72
Retroelements	LTR elements	BEL/Pao	35	6430	0.01
Retroelements	LTR elements	Ty1/Copia	734	897080	1.65
Retroelements	LTR elements	Gypsy/DIRS1	874	1824229	3.35
Retroelements	LTR elements	Retroviral	0	0	0
DNA transposons	—	Total DNA transposons	292	237335	0.44
DNA transposons	—	hobo-Activator	0	0	0
DNA transposons	—	Tc1-IS630-Pogo	0	0	0
DNA transposons	—	En-Spm	0	0	0
DNA transposons	—	MULE-MuDR	0	0	0
DNA transposons	—	PiggyBac	0	0	0
DNA transposons	—	Tourist/Harbinger	0	0	0
DNA transposons	—	Other (Mirage, P-element, Transib)	0	0	0
Rolling-circles	—	Rolling-circles	32	67931	0.12
Unclassified	—	Unclassified	7235	3772279	6.93
Total interspersed repeats	—	Total interspersed repeats	—	7705704	14.16
Small RNA	—	Small RNA	73	206431	0.38
Satellites	—	Satellites	0	0	0
Simple repeats	—	Simple repeats	6538	304399	0.56
Low complexity	—	Low complexity	1338	76104	0.14
Total masked	—	Total masked	—	8360569	15.36
